# Exploring how brain health strategy training informs the future of work

**DOI:** 10.3389/fpsyg.2023.1175652

**Published:** 2023-09-12

**Authors:** Jennifer Zientz, Jeffrey S. Spence, Susan Sung Eun Chung, Upali Nanda, Sandra Bond Chapman

**Affiliations:** ^1^Center for BrainHealth^®^, School of Behavioral and Brain Sciences, The University of Texas at Dallas, Dallas, TX, United States; ^2^HKS Inc., Dallas, TX, United States

**Keywords:** brain training, burnout, workplace, mental health, brain health, productivity, hybrid work

## Abstract

**Introduction:**

The workplace typically affords one of the longest periods for continued brain health growth. Brain health is defined by the World Health Organization (WHO) as the promotion of optimal brain development, cognitive health, and well-being across the life course, which we expanded to also include connectedness to people and purpose. This work was motivated by prior work showing individuals, outside of an aggregate setting, benefitted from training as measured by significant performance gains on a holistic BrainHealth Index and its factors (i.e., clarity, connectedness, emotional balance). The current research was conducted during the changing remote work practices emerging post-pandemic to test whether a capacity-building training would be associated with significant gains on measures of brain health and components of burnout. The study also tested the influence of utilization of training modules and days in office for individuals to inform workplace practices.

**Methods:**

We investigated whether 193 individuals across a firm’s sites would improve on measures of brain health and burnout from micro-delivery of online tactical brain health strategies, combined with two individualized coaching sessions, and practical exercises related to work and personal life, over a six-month period. Brain health was measured using an evidenced-based measure (BrainHealth™ Index) with its components (clarity, connectedness, emotional balance) consistent with the WHO definition. Burnout was measured using the Maslach Burnout Inventory Human Services Survey. Days in office were determined by access to digital workplace applications from the firm’s network. Regression analyses were used to assess relationships between change in BrainHealth factors and change in components of the Maslach Burnout Inventory.

**Results:**

Results at posttest indicated that 75% of the individuals showed gains on a composite BrainHealth Index and across all three composite factors contributing to brain health. Benefits were directly tied to training utilization such that those who completed the core modules showed the greatest gains. The current results also found an association between gains on both the connectedness and emotional balance brain health factors and reduced on burnout components of occupational exhaustion and depersonalization towards one’s workplace. We found that fewer days in the office were associated with greater gains in the clarity factor, but not for connectedness and emotional balance.

**Discussion:**

These results support the value of a proactive, capacity-building training to benefit all employees to complement the more widespread limited offerings that address a smaller segment who need mental illness assistance programs. The future of work may be informed by corporate investment in focused efforts to boost collective brain capital through a human-centered, capacity-building approach. Efforts are underway to uncover the value of better brain health, i.e., *Brainomics©* - which includes economic, societal, and individual benefits.

## 1. Introduction

The dramatic disruptions in workforce policies and individual preferences that ensued from the pandemic opened new opportunities to rethink and reimagine the workplace culture. Considerations of people, place, purpose, and productivity are top of mind and focus for corporate leaders. Long-standing and widely held perspectives have been upended regarding where and how people perform best and which settings support optimal individual versus collaborative work, e.g., working remotely (WR) versus In-Office (Rozentals, 2022). Most organizations remain in a fluid situation with continued uncertainty as to what works best for both employees and companies to thrive and how the betterment of one benefits the other. Companies are scrambling to navigate what changes are needed immediately and in the long term to attract, train, and retain employees within a culture that grows talent, optimizes wellbeing and mitigates burnout.

The time is right to better understand and investigate promising protocols and outcomes that may inform the changing landscape of workplace policies. Boosting brain skills for individuals is an effort that is gaining momentum, as a possible path to rebuild a thriving workforce, economy, and society (Greene, 2021; Smith et al., 2021). The brain drives all that we do, think, create, overcome, solve, and connect meaningfully to others (Chapman et al., 2022). Brain skills support teamwork, leadership, innovation and adaptability (Greene, 2021).

One clear need is to identify and test the effectiveness of programs that could enable employees to increase their personal resources to better thrive in the workplace while simultaneously achieving the organization goals related to productivity and economic viability (Ho and Chan, 2022). More precisely, Ho and colleague (2022) state that, “pursuit of a happy and satisfied workforce is an important goal not only as an end in itself but also as a means to employer’s desired productivity level.” There is a major need for more evidence to guide companies on how to make meaningful progress toward this goal. The present study attempts to fill this void by investigating whether a workplace-offered personal resource capacity-building training would benefit employees’ brain health and mitigate burnout whether working remote or in office.

### 1.1. Individual capacity and resource restitution

Considerable attention is directed toward employee assistance programs that address the growing mental health crises and aggravated burnout. The relationship between mental health and burnout is complex and it is unknown whether addressing mental health problems will also serve to mitigate burnout. Mental illness is one of the greatest cost burdens for a company in terms of days of work lost, medical expenses, and low productivity while dealing with depressions, stress, and anxiety (Sapien Labs, 2020; Minor, 2021; Witters and Agrawal, 2022). Prior to COVID-19, disorders of the brain were estimated to cost the global economy at least $2.5tn in lost productivity every year (Eyre et al., 2020). As a result, many workplaces are expanding programs focused on diagnosing and treating mental illness such as depression, anxiety, stress, and suicidality, but the problem is still expanding.

Burnout is defined by the World Health Organization (2019) and can be measured by the scientifically validated Maslach Burnout Inventory (Maslach and Jackson, 1981). WHO’s definition of burnout is characterized by three components: (1) feelings of energy depletion or exhaustion, (2) increased mental distance from one’s job, or feelings of negativism or cynicism related to one’s job, and (3) reduced professional efficacy (Maslach and Leiter, 2021). Post-pandemic, employers have witnessed growing complaints of burnout and distress across many industries with the most commonly associated cause being prolonged stress (Bakker and Demerouti, 2007). The authors of the Maslach Burnout Inventory warn that the rating scale was never developed to clinically diagnose burnout as a personal disability (Maslach and Leiter, 2021). Nor was it developed as a single-pronged solution to be addressed with a focused intervention. Rather, its developers stated the utility comes from being combined with other personally salient information to guide leaders to make changes and design healthier workplace practices in which employees will thrive.

A resource-restitution approach that offers diagnosis and treatment for mental health problems or burnout, while vital, may be insufficient to get ahead of and address the possibility of programs to help workers thrive through a wide range of solutions and actions. As such, training programs to promote better workforce health outcomes for the individual and the company are being sought. Some efforts have examined the contribution of mindfulness in predicting and perhaps mitigating burnout in the workplace (Taylor and Millear, 2016). For example, Taylor and colleague (2016) suggested that overall mindfulness, shown to be a unique personal trait, may provide a personal internal resource that could buffer burnout complaints. They concluded that mindfulness may be a way to replenish your mental resources to mitigate against burnout.

According to one of the nation’s leading mental health experts, mental health is a medical problem, but the solutions are not just medical – they are social, environmental, political, and even spiritual (Insel, 2022). Insel claims we are not in a mental illness crisis but rather in a crisis of mental *health care*. We agree with this viewpoint and add that the solutions are also cognitive (Venza et al., 2016; Han et al., 2018; Chapman et al., 2021; Laane et al., 2022). We propose that capacity-building protocols may help to protect against and even intercept mental health issues by giving individuals the tools to be agents of change by drawing upon their internal resources.

### 1.2. Individual capacity-and resource-building approach

Support for a capacity-building approach, in which individuals learn ways to grow and utilize their brain skills in more efficient and effective ways continuously, is implicated in a recent study which examined the dynamic relationships across individual and organizational level factors that impact workplace health and culture (Al-Jubari et al., 2022; Ho and Chan, 2022). Findings from a prospective one-year, non-intervention, observational study reported that both (1) the personal resources of an *individual* and the (2) degree of perceived *organization* support were foundational to ‘flourishing’ in the workplace (Ho and Chan, 2022). Flourishing was defined as maximizing ones’ potential and living in the fullest to achieve optimal psychosocial functioning.

Ho and Chan (2022) conceptualized personal resources as a reservoir of capacities that can be acquired and strengthened over time and include these three domains: conditions (e.g., social support), personal characteristics (e.g., self-efficacy), and energies (e.g., effort). These researchers found that when perceived organizational support was strong, employees endorsed higher ratings of their personal resources, specifically on ratings of hope, efficacy, resilience, and optimism. These two individual and organizational domains (personal resources and perceived organization support) worked in concert to mediate higher self-ratings on two separate measures of flourishing. These efforts implicated a dual role of individual resources (e.g., brain skills) and perceived organization support as contributing sources to individual ratings of flourishing.

This current study represents an early attempt to examine the impact of a capacity-building approach to a workplace by deploying science-backed brain health measurements and training protocols. To date, efforts to strengthen brain health has received little to no attention in the workplace. This lack of effort is largely due to the limited access to measurements and proven protocols shown to improve brain health in healthy adults.

### 1.3. Framework for a brain health approach

#### 1.3.1. Definition

Brain health is defined by the World Health Organization as the *continual* promotion of optimal brain development/fitness across the lifespan, cognitive health, and emotional wellbeing (World Health Organization, 2022) with our addition of connectedness to people and purpose. Brain health is conceived as a superordinate category of health; mental health and social–emotional health/connectedness are subordinate and subsumed under brain health (Chapman et al., 2022).

Workplace leadership is becoming increasingly aware that human capital is an organization’s greatest and most valuable asset and investment in this 21st century (Greene, 2021; Smith et al., 2021). Research shows these brain skills can be reinforced and expanded to contribute to one’s ability to draw upon and challenge up brain resources to meet the demands at hand (Chapman et al., 2016). These broad-based brain skills are pivotal to calibrating mental effort and energy, agility in thinking, speed of learning, relating to others with empathy, and finding greater personal purpose and fulfillment outside the office (Chapman et al., 2022). The future of work is in urgent need of programming that offers effective ways to boost brain skills from a holistic, broad-based perspective to complement programs offering assistance for medical concerns, such as depression.

#### 1.3.2. Brain health measurement and training

In prior work, we have shown that participants, outside of aggregate settings such as workplace or group contexts, significantly improved their brain skills across multiple domains of intellect, wellbeing, social connectedness, and real-life responsibilities (Chapman et al., 2015; Venza et al., 2016; Chapman et al., 2021; Laane et al., 2022). This work has led to development and testing of validated BrainHealth Index that can be used to evaluate improved brain health over time, regardless of intervention. The BrainHealth Index metrics have been associated with significant changes in key brain networks following a brain health training program (Chapman et al., 2015; Gallen et al., 2016; Motes et al., 2018). Our conceptual framework for brain health training is guided by principles of neuroplasticity and motivated by extant evidence that healthy individuals can benefit from brain health strategies, giving them the tools to take charge of dealing with the daily challenges they face, both professional and personal. Our research and others revealed that brain health training improves reasoning, innovation, wellbeing, social connectedness, daily life activities and converging neural changes (Chapman and Mudar, 2014; Vas et al., 2016).

### 1.4. Objectives

The present study extends our earlier work with individuals who were not recruited from a functionally related group, to examine whether participants recruited from a single organization would show similar benefits. In other words, would access to a workplace-wide brain health training be associated with significant measurable gains in individual brain capacities? We proposed that a proactive brain health training within a company may provide a supportive culture to reinforce utilization of healthy mental habits through a common language with greater efficacy than can be achieved with one-off individual trainings with perhaps a transfer benefit to reduce burnout. Given the ambiguity of workplace decisions about remote work, we were particularly interested in whether brain health training had a differential impact based on place of work, i.e., average days/week working remote versus in-office. We predicted that those who averaged 2–3 days/week in the office would show the greatest gains on the BrainHealth Index and reduced burnout based on recent findings (Choudhury et al., 2022).

This study investigated whether 193 individuals (78 who completed post assessments – Time 2) in a global architecture and design firm would benefit from micro-delivery of tactical brain health strategies combined with individualized coaching and practical exercises that apply to work and personal life. We addressed these specific questions:

1. Did brain health training benefit individuals across multiple sites of a single architecture and design firm as measured by performance gains on a validated BrainHealth Index (the integrated score and the three factors scores of clarity, connectedness, and emotional balance)?
2. Did gains on the composite BrainHealth Index and the three factors (clarity, connectedness, and emotional balance) correspond to significant changes on the Maslach Burnout Inventory Human Services Survey’s three components (emotional exhaustion, depersonalization, and personal accomplishment)?
3. Did average days per week working remote (WR)/in-office relate to changes in the BrainHealth Index and/or the Maslach Burnout Inventory Human Services Survey components?

Our research over the past two decades has taken a capacity-building, proactive approach to evaluate whether promoting better brain health can avoid stigma and get ahead of problems before they become clinically significant rather than a sole focus on diagnosing and addressing problems. Prior work has shown that improved brain health can have a spill-over benefit to wellbeing and social connections (Vas et al., 2016; Laane et al., 2022). We wanted to examine the impact of a workplace-wide program that focused on boosting brain health skills to benefit all individuals, instead of a subset with concerns. The workplace is often the longest-term time and place where our brain skills are further developed or perhaps diminished. The average knowledge worker typically spends more of their brain years in the workforce than years of education. This study is relevant to explore the vast opportunities to promote continual development of optimal brain skills at our professional work to add to the years of education where we acknowledge brain skills are built.

## 2. Methods

This study adhered to the standards of The University of Texas at Dallas Institutional Review Board. Participants were informed about the protocol prior to obtaining electronic informed consent.

### 2.1. Recruitment

Participants were employees of a large global architecture and design firm. They were recruited during a sign-up period following a firmwide presentation on the importance and benefits of brain health. Employees who had completed at least one self-reported pulse survey that was initiated by the firm at the start of the COVID-19 pandemic were eligible. The pulse surveys were conducted during the time period of March 2020 through August 2021 and included validated measures related to work behaviors and work conditions, such as days in office. Inclusion criteria for this study consisted of being 18 years of age or older, able to access the internet through computer, tablet or smartphone, and being a proficient English speaker. Exclusion criteria consisted of having a diagnosed neurological disorder or disease or uncontrolled psychiatric disorder. Because this was a workplace study, participants were not excluded for general health risk factors.

Two hundred fifteen team members were selected to participate, and of those, 193 consented and completed baseline assessment. Of those, 164 engaged in virtual coaching session, and 101 completed a minimum of the core training microlearning units. Seventy-eight participants took the post-training assessment, and of those, 28 engaged in a virtual coaching session. Attrition was reportedly due to being too busy and/or not having time to engage in the training or complete the post-training assessment (Table 1).

**Table 1:** Demographics.

Age	Mean	SD	Min	Max
	43.9	11.3	25	71
Education	Male	Female	Total	
<Bachelor’s	4	6	10	
Bachelor’s	27	62	89	
>Bachelor’s	38	56	94	
Total	69	124	193	

### 2.2. Study protocol

This study consisted of online assessments, virtual coaching, and online training over a six-month period.

Participants completed online assessment measures, including metrics of brain health and burnout. Within 2 weeks of completion of the online assessments (Time 1), participants received their BrainHealth Index score and had a virtual coaching session with a BrainHealth coach to debrief on their results and get individualized recommendations for interacting with the online training content based on their performance. BrainHealth coaches were masters-level clinicians with extensive experience with both the assessment metrics and training content. Participants were directed to begin self-paced online training modules, which consisted of microlearning units lasting approximately 5–10 min per day. At the six-month milestone, participants completed the online assessment measures for a second time (Time 2) and again had a virtual coaching session with a BrainHealth coach to debrief on their results and get recommendations for ongoing use of the strategies learned in the online training.

### 2.3. Metrics

Participants completed a series of online assessments that measured key domains of brain health and aspects of burnout. The firm used server data to calculate the average number of days participants spent working in the office.

### 2.4. BrainHealth™ index

Participants completed a multi-dimensional assessment of brain health and performance, which is a composite of 22 gold standard measures to capture the rich multi-dimensionality and growth potential of our complex brain skills (Chapman et al., 2021). The index was designed to measure the continual development of cognitive health, wellbeing and connectedness to people and purpose across the lifespan and is being validated by a range of MRI neural markers applied repeatedly over time to healthy populations ranging in age from 18–95 years (Chapman et al., 2022). The BrainHealth Index was not developed to establish a diagnostic label, but rather to motivate individuals to maintain and strengthen their brain capacities and resources whatever the starting point. The Index has four scores. The key one is a composite or global BrainHealth Index score representing one’s integrated brain capacities that work in concert to support their daily mental tasks/activities. From the Index, three validated Factors scores are provided to convey to individuals how they can take steps to continually expand their overall brain performance through these multiple paths. The factors are: (1) *Clarity* (cognitive health), (2) *Connectedness to people and purpose* (social health), and *Emotional balance* (wellbeing) (Table 2).

**Table 2:** Individual measures that comprise the BrainHealth Index and factors.

Factor	Measure	Reference
Clarity	Strategic attention	Visual Selective Learning Task (Hanten et al., 2007)
	Abstraction	Proverb interpretation (developed at the Center for BrainHealth)
	Reasoning	Test of Strategic Learning (TOSL) (Vas et al., 2015)
	*Synthesis*	High-level summary of text
	*Interpretation*	Take-home messages/interpretations from text
	*Memory*	Memory for text details (free and cued/elaborated recall)
	Sleep	Pittsburgh Sleep Quality Index (PSQI) (Buysse et al., 1989)
	Compassion	Adapted from the Light Triad Scale (Strauss et al., 2016; Johnson, 2018)
Emotional balance	Depression Anxiety Stress	Depression Anxiety Stress Scale (DASS-21) (Lovibond and Lovibond, 1995)
	Sleep	Pittsburgh Sleep Quality Index (PSQI) (Buysse et al., 1989)
Connectedness	Activities	Engagement in Meaningful Activities Survey (EMAS) (Eakman, 2011)
	Happiness	Oxford Happiness Questionnaire (OHQ) (Hills and Argyle, 2002)
	Social support	Social Support Survey Index (Sherbourne and Stewart, 1991)
	Resilience	Connor-Davidson Resilience Scale (Connor and Davidson, 2003)
	Life satisfaction	Quality of Life Scale (Burckhardt and Anderson, 2003)
	Social engagement	Social BrainHealth Scale (developed at the Center for BrainHealth)
	Compassion	Adapted from the Light Triad Scale (Strauss et al., 2016; Johnson, 2018)

Sleep and compassion measures loaded significantly onto more than one factor.

### 2.5. Maslach Burnout Inventory

Participants also completed the Maslach Burnout Inventory Human Services Survey (Maslach and Jackson, 1981). This version of the inventory assesses three key aspects to burnout, which are labeled: emotional exhaustion, depersonalization, and personal accomplishment. Emotional exhaustion is characterized by feelings of energy depletion from an individual’s work and being emotionally overextended. Depersonalization is characterized by an unfeeling and impersonal response toward the recipients of one’s work. Personal accomplishment is characterized by feeling competent and achieving success in working with others. The survey includes 22 statements to which participants respond with the level of frequency they experience each. Nine of the statements target emotional exhaustion. Examples include: ‘I feel emotionally drained from my work’ and ‘Working with people all day is really a strain for me.’ Five of the statements target feelings of depersonalization, with statements such as: ‘I’ve become more callous toward people since I took this job’ and ‘I do not really care what happens to others.’ Eight of the statements relate to personal accomplishment. Examples of statements include: ‘I have accomplished many worthwhile things in this job’ and ‘In my work, I deal with emotional problems very calmly.’ Ratings of frequency are 0–6, with 0 being ‘never’ and 6 being ‘every day’. This Human Services Survey was selected over the Maslach Burnout Inventory General Survey version (Maslach and Jackson, 1986) because it has been more extensively used across a variety of professional fields that focus on helping individuals to live better lives, which is the primary goal of the architecture and design firm.

### 2.6. Average days/week in office

Participants accessing digital workplace applications from the firm’s network, as opposed to a remote internet connection, logs data in an authentication application. The data is recorded each day as a value of 1 for working in one of the firm’s multiple office locations and 0 for working elsewhere (e.g., home, client site, travel, etc.). The result of these logs provides an estimate of average daily office attendance over a period of time with an estimated margin of error of 5–10 percent. The data was collected for study participants for two separate six-week periods: February 21, 2022 to April 1, 2022 for Time 1 and July 1, 2022 to August 19, 2022 for Time 2. This provides the value (range of 0 to 5) for average days in an office per week across each such period.

### 2.7. Coaching

Participants were offered the opportunity to engage one-on-one with a BrainHealth coach at Time 1 and Time 2. Each coaching session was 30 min and was done via video conference. The purpose of the coaching sessions was to debrief on the participant’s BrainHealth Index, set expectations and provide direction for ways to engage with the online training content, and to assist participants in setting personal goals utilizing the strategies presented in the online training.

### 2.8. Training

All participants gained access to an online dashboard that delivered cognitive training in microlearning sessions (Chapman et al., 2022). The training protocol is comprised of evidence-based strategy learning, which has been shown through clinical trials to positively impact neurocognitive and real-life function (Chapman and Mudar, 2014; Chapman et al., 2015). The protocol, Strategic Memory Advanced Reasoning Training (SMART), was developed by Center for BrainHealth neuroscientists and clinicians based on 30 years of study. SMART has been shown to positively impact areas of cognition (strategic attention, integrated reasoning, innovation and memory), wellbeing (reduced stress, depression and anxiety), and real-life function (improved quality of life, initiation of social engagement and complexity of life work). Changes in these areas have been correlated with significant neural changes, including neural connectivity, cerebral blood flow, and neural efficiency (Chapman et al., 2015, 2016, 2017; Gallen et al., 2016; Han et al., 2017, 2018; Motes et al., 2018).

Training was self-paced and consisted of 5–10 min daily micro-units teaching tactical brain strategies that are applicable to both professional and personal life circumstances. The training units provide strategy education, rationale in terms of brain health literacy, and opportunity for personal reflection and application to a participant’s life.

SMART units include strategic attention, integrated reasoning, and innovation (each of which taught a set of three strategies), as well as application practice. Strategic attention strategies help participants down-select information, thereby reducing information overload (via filtering what is unnecessary) and enabling a stronger ability to focus on key data and ideas that are critical to task at hand (versus all data). Integrated reasoning strategies emphasize the ability to abstract meanings and concepts from key data and ideas and apply them to a broader context, underscoring the relevance of seemingly disparate ideas to one another on a more global level. Innovation strategies encourage participants to be flexible thinkers and to generate multiple possibilities that exist beyond the standard or initial solution, to seek different perspectives outside of their own, to recognize mistakes that are learning blocks and to challenge their status quo in seeking curiosity. The application unit provided real-life examples of how to use the strategies in a cohesive way. These strategy-based modules/units comprised the core training.

After completing the SMART modules, participants continued with online learning about stress solutions and the science of sleep. Stress solutions tied the SMART strategies to stress management techniques to help build a resilient mindset. The stress solutions modules also provided information on healthy lifestyle choices, such as diet and exercise, as well as provided information on mindful meditation, all of which have been empirically shown to reduce stress. The sleep module provided participants with information on the science of sleep and sleep cycles, as well as sleep hygiene tips to help improve the quality and quantity of sleep (Table 3).

**Table 3:** Description of self-paced training modules.

Training module	Description	No. of units/ Total time
1. SMART 01*	Provides strategies and interactive activities on how to block irrelevant information and focus on key priorities and data (strategic attention). *Example: Organize your day to accomplish significant tasks – each day prioritize the top two tasks that require the most deeper level thinking.*	5 units/45 min
2. SMART 02*	Provides strategies and interactive activities on how to abstract big-picture concepts from information to better inform real life decisions (integrated reasoning). *Example: Extract key concepts from incoming information* vs. *trying to onboard and remember everything.*	4 units/35 min
3. SMART 03*	Provides strategies and interactive activities on how to generate multiple and diverse solutions/perspectives to strengthen mental flexibility (innovation). Example: Identify multiple alternative perspectives/ideas on discordant issues.	6 units/35 min
4. SMART 04*	Provides real-life application scenarios where participants can practice dynamic implementation of the strategies from SMART 01–03 (strategic attention, integrated reasoning, innovation) in a cohesive manner. *Example: Think about and prepare to ask your boss for a raise* (*considering your accomplishments, impact those accomplishments have had on the organization*, etc.).	6 units/45 min
5. Stress solutions 01	Presents physiological and neurological response to stress, as well as cognitive strategies linked with SMART to manage and reframe stressors. *Example: Reframe your perception of your response to a difficult situation from anxiety to excitement.*	5 units/40 min
6. Stress solutions 02	Provides accessible techniques to help “recharge your battery” in terms of stress or fatigue, as well as education on lifestyle factors that can positively impact overall health. *Example: Take several short breaks throughout your day.*	4 units/30 min
7. Stress solutions 03	Provides research on the benefits of mindfulness, meditation, and healthy sleep habits, as well as practical tips on how to practice each one (linking with SMART). Example: Participate in a meditation exercise.	5 units/45 min
8. Sleep	Presents research on the science behind sleep, the brain impact of poor sleep, and practical tips for improving one’s sleep habits.	16 units/75 min
Total time		350 min

*Units 1–4 are considered the core training, as they provide the bulk of cognitive strategies.

### 2.9. Statistical analysis

Associations between six-month change in BrainHealth Index factors and six-month change in the Maslach Burnout Inventory components or average days per week in office were tested via regression analyses. Since these measurements were taken from participants across sites at the architecture and design firm, we included site as a factor in the regression models, as well as its interaction with the component scores, to account for site variability in the regression estimates. There were a total of 12 regression coefficient tests — 3 Index factors x (3 burnout components +1 measure of days-in-office). To account for the multiple testing, we controlled the false discovery rate (FDR) at the level of 0.05. All *p*-values that satisfy the FDR criterion are indicated by asterisks in the Results tables below.

## 3. Results

### 3.1. Attrition

The attrition rate was approximately 61% (117/193), leaving a total sample size of 76 having complete Time 1 and Time 2 data. We compared baseline characteristics between the dropout sample and non-dropout sample to assess potential differences. Table 4 shows the summaries for gender, age and several assessments, including the BrainHealth Index, the emotional exhaustion component of the Maslach Burnout Inventory and average days in office per week. Both samples show similar means and percentages for all baseline characteristics, which is evidence that there did not exist systematic reasons for dropout. All analyses, therefore, were based on the sample with complete data at both time points.

**Table 4:** Baseline characteristics of dropout and non-dropout samples.

	Dropout*	Non-Dropout*	Odds Ratio	*p*-value
Gender	62.4	67.1	1.23	0.541
	Dropout**	Non-Dropout**	*t*-statistic	*p*-value
Age	45.2 (1.1)	43.5 (1.3)	0.99	0.326
BHI	478.3 (7.2)	487.0 (8.7)	−0.76	0.449
Exhaustion	24.1 (1.1)	22.5 (1.3)	0.94	0.351
Days in office	1.38 (0.01)	1.41 (0.02)	−0.11	0.912

*% Female; **Mean (se).

### 3.2. Brain health indices

**Figure 1:**
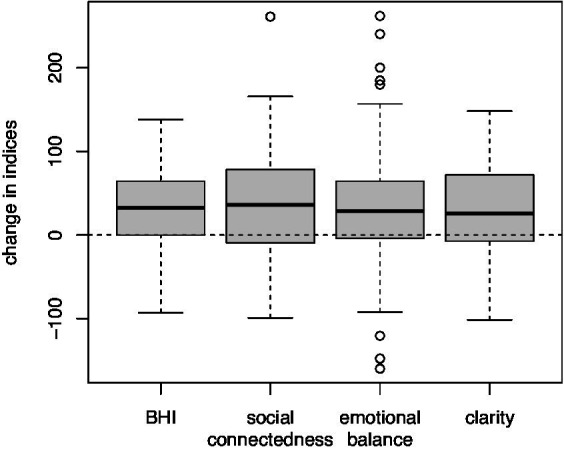
Boxplot summaries of the distributions of change in the composite BrainHealth Index (BHI) and the three individual BrainHealth factors. All indices (i.e., BHI and factor scores) gained between 25.3 and 36.8 units on average, while nearly 75% of participants showed improvements 6 months after their baseline assessments.

There was substantial gain between baseline index scores and scores 6 months post-training for all of the index measures with nearly 75% of participants showing improvement. Figure 1 shows boxplot summaries of the gains for each of the factor indices, as well as the overall BrainHealth Index (BHI). The connectedness factor, in particular, had the largest average gain (36.8 units, an effect size of 0.62), while the clarity index showed an average improvement of 25.3 units (effect size of 0.43.) Emotional balance and the BHI gained 32.3 and 31.5 units (effect sizes 0.54 and 0.53, respectively, see Table 5 for summary statistics.) Post-training gains did not depend on age or gender. That is, the change in each of the index measures was similar for both men and women, and also similar across the age range.

**Table 5:** Change in composite BrainHealth Index and BrainHealth factors following training.

	Estimate	Std error	*t*-statistic	*p*-value	Effect size (d)
Change in BHI	31.5	5.2	6.02	<0.001	0.53
Change in social connectedness	36.8	7.4	4.97	<0.001	0.62
Change in emotional balance	32.3	8.7	3.72	<0.001	0.54
Change in clarity	25.3	6.7	3.77	<0.001	0.43

### 3.3. Training utilization

**Figure 2:**
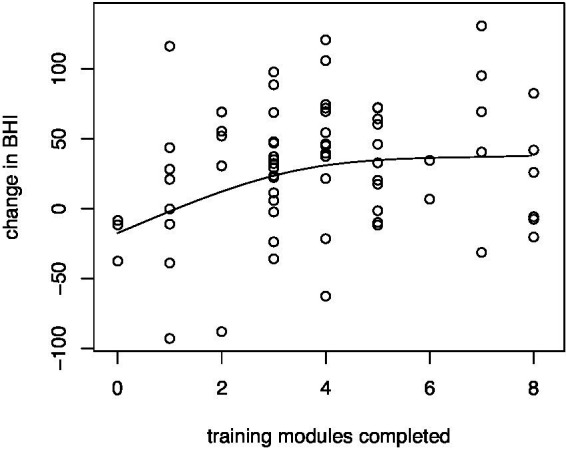
Regression of change in the composite BrainHealth Index on training utilization. Improvements on the BHI increased linearly – an average of 52.3 units – as participants completed the core training modules (i.e., modules 1–4), beyond which there were no further improvements.

Participants completed a variable number of training modules prior to their Time 2 measurements, which allowed an assessment of the effect of training utilization on change in the BHI. Figure 2 shows a regression of BHI change on the number of training modules completed using a 3-degree-of-freedom natural spline basis. For those who did not complete any of the modules, the average change in the BHI was-17.4. However, the change in the BHI improved linearly to an average 34.9 for those who completed the core training, a significant difference of 52.3 units (*p* = 0.017). Beyond the core training modules, there was no further improvement in the BHI.

There was a similar linear improvement, an average decrease of 6.2 units, in the exhaustion component of the Maslach Burnout Inventory between those who did not complete any of the training modules and those who completed the core training, as well as a similar plateau beyond the core training. However, the decrease in the exhaustion scores was not statistically significant (*p* = 0.14).

### 3.4. Relationship of brain health indices with the Maslach Burnout Inventory

**Figure 3:**
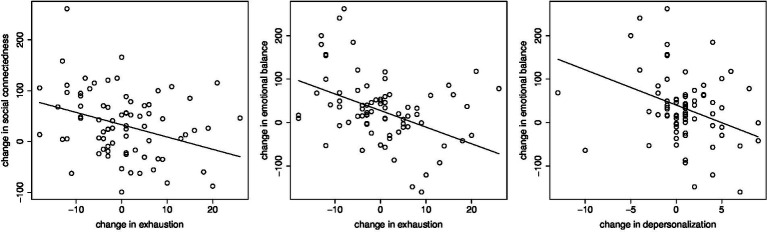
Regression of change in the social connectedness factor (left panel) and change in the emotional balance factor (center panel) on change in the exhaustion component of the Maslach Burnout Inventory; right panel shows regression of change in the emotional balance factor on change in the depersonalization component of the Maslach Burnout Inventory. Improvements on the burnout scales in each panel were associated with improvements in the BrainHealth factor scores.

We did not observe significant change in any of the components of the Maslach Burnout Inventory Human Services Survey — emotional exhaustion, depersonalization, and personal accomplishment — 6 months following the training. However, there were strong linear relationships between the change in the exhaustion component and change in both the social connectedness (*p* = 0.003) and emotional balance (*p* < 0.001) indices. Additionally, we found a linear relationship between the change in the depersonalization component and change in the emotional balance (*p* = 0.016) index. Figure 3 shows that improvement in the social connectedness index was associated with significant reductions or improvement in the exhaustion component, while improvement in the emotional balance index was associated with reductions or improvement in both the exhaustion component and the depersonalization score. Conversely, declines in both social connectedness and emotional balance were associated with significant increases or worsening of rating for the exhaustion component, and declines in the emotional balance index was associated with increases or worsening of rating for the depersonalization score. We did not find any such association between the clarity index and either the exhaustion component or the depersonalization component of the burnout inventory (see Tables 6, 7 for summary statistics), nor did we find any associations between the third component of the burnout inventory, personal accomplishment, with any of the brain health index measures.

**Table 6:** Regression of change in BrainHealth factors on change in the emotional exhaustion component of the Maslach Burnout Inventory.

	Estimate	Standardized estimate	Std error	*t*-statistic	*p*-value
Change in social connectedness	−2.42	−0.34	0.78	−3.08	0.003*
Change in emotional balance	−3.82	−0.46	0.94	−4.06	<0.001*
Change in clarity	−1.13	−0.18	0.79	−1.42	0.160

*FDR = 0.05.

**Table 7:** Regression of change in BrainHealth factors on change in the depersonalization component of the Maslach Burnout Inventory.

	Estimate	Standardized estimate	Std Error	*t*-statistic	*p*-value
Change in social connectedness	−3.26	−0.46	2.09	1.15	0.256
Change in emotional balance	−8.11	−0.98	2.52	2.47	0.016*
Change in clarity	−0.60	−0.09	1.98	0.23	0.817

*FDR = 0.05.

### 3.5. Days in office

**Figure 4:**
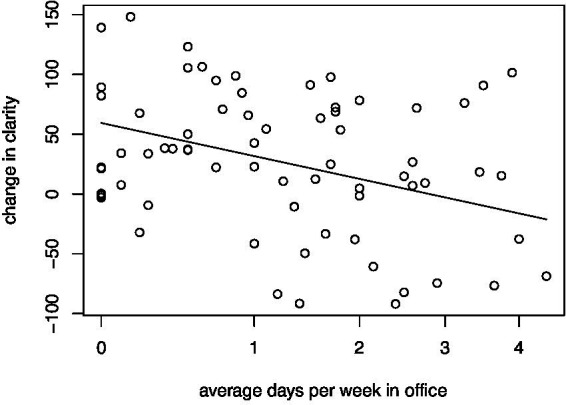
Regression of change in the clarity factor on average number of days per week in office. A significant negative relationship exists such that a large gain in clarity is associated with no days in office. As the number of average days in office increases, the change in clarity decreases. For those working an average 4 days in office, there is no average gain in the clarity index. Average days per week in office is on a shifted square root scale.

There was no significant change in the average days per week in office between baseline measures and those taken 6 months post-training. However, Figure 4 shows that the average number of days per week in office was negatively associated with change in the clarity index (*p* = 0.010). An average 50-point gain in clarity was observed for those who did not work in office; whereas no average change in clarity was observed for those who worked approximately 4 days per week in office. Table 8 shows the summary statistics for each index of brain health. We did not find any associations between average days per week in office and the other brain health indices, nor did we find any associations between average days per week in office and the component scores of the Maslach Burnout Inventory.

**Table 8:** Regression of change in BrainHealth factors on the average days per week in office.

	Estimate	Standardized estimate	Std error	*t*-statistic	*p*-value
Change in social connectedness	−15.3	−0.11	23.5	−0.65	0.517
Change in emotional balance	7.7	0.04	30.7	0.25	0.803
Change in clarity	−53.5	−0.39	20.1	−2.66	0.010*

*FDR = 0.05.

## 4. Discussion/conclusion

This study represents an early effort to examine the benefits of a human-centric, brain health training program to impact individuals in a workplace in this turbulent post-pandemic era. The key goals were to evaluate whether individuals across sites from a single company would personally benefit from SMART training as manifested by significant gains on a validated metric of brain health, i.e., the BrainHealth Index. Secondly, we addressed whether better brain health was associated with reduced burnout, as measured by the Maslach Burnout Inventory Human Services Survey components. A third goal explored the impact of hybrid work on these measures, by tracking days WR and In-Office. Specifically, we examined whether gains on brain health measures or changes in burnout components were influenced by average number of days in the office over a six-month period during the current study.

### 4.1. Enhancing brain health in the workplace

The most important finding was improved brain performance for individuals across corporate sites as manifested by gains on a composite BrainHealth Index following training. This promising outcome of increased brain performance was achieved during a time of profound change and high anxiety across all workplaces. As stated in the Methods, the BrainHealth Index provides a composite score across multiple dimensions, which are requisite to adapting to meet the changing challenges of the workplace and personal life contexts. The Index was developed not only to measure baseline performance but also to track changes in brain skills, either maintained, showed gains, or revealed losses over time. We interpret this finding to suggest that the majority of individuals increased their brain performance by taking advantage of the core training which guided them in ways to adopt brain healthy habits to utilize their own mental resources more effectively. The training reinforces self-agency to encourage individuals to uncover ways to adapt to changes and uncertainty as a curiosity challenge throughout the day.

The pattern of improved performance after training is consistent with prior results from a population-based study of healthy individuals, ages 18–87 from disparate places and work contexts (Chapman et al., 2022). That is, we found that the average composite gain was comparable to that achieved with individuals from our previous research not in aggregate settings. The gains in both present and the prior studies are attributed to benefits from the training and not to practice effects. The inadequacy of a practice effect explanation is supported by prior evidence that people who took the Index at the same designated two time periods but did little to none of the training, did *not* show significant gains. We had anticipated that participants in an aggregate workplace setting would show greater gains than dispersed individuals without common connection to reinforce strategies. It was reassuring to find that individuals benefitted whether in aggregate or dispersed contexts. Nonetheless, we propose that the failure to find a larger benefit in an aggregate setting versus dispersed individuals may require larger number of participants within a single office. Future work will focus on whether even greater gains can be achieved within a single workplace setting that seeks to reinforce brain strategy application within teams.

Another major finding was significant improvement on the three core factors comprising the composite BrainHealth Index, e.g., clarity, connectedness to people and purpose, and emotional balance. Each of these three factors provide individuals multiple paths to improve their ability to calibrate and reset their mental effort throughout the day. These three factors represent the same dimensions set forth in the definition of brain health by the World Health Organization (World Health Organization, 2022) with our addition of connectedness to people and purpose. This finding adds further support for our claim that people have different brain skills from which to draw to compensate for vulnerabilities to continue to maintain brain health from day to day (Chapman et al., 2022). For example, on 1 day individuals may expand their innovative problem-solving skills or reduce information overload, while other times draw from their social adeptness or be motivated by a meaningful purpose to fuel their efforts, or perhaps adopt strategies to maintain calm with emotional balance rather than emotional reactivity in the midst of uncertainty and change. During the brain health training, individuals learn strategies that they could apply in their professional and personal lives. They were provided an array of practical exercises to experience how they can become more agile and intentional to adapt to daily demands across both contexts.

### 4.2. Mitigating burnout through better brain health

We expected to find meaningful relationships between gains on the BrainHealth Index and changes on the Maslach Burnout Inventory components, based on prior evidence that the BrainHealth Index is a measure of one’s dynamic ability to flourish (Neill and Nevin, 2021; Chapman et al., 2022). As reported in the Results section, these postulations were supported. Our findings support the view that linkages between wellbeing and depersonalization do not appear to represent a trait/static pattern. We found mutual change benefits in two facets of brain health and burnout components following the brain health training. Specifically, as individuals’ *connectedness* to people and purpose and their *emotional balance/wellbeing* improved following the brain health training, there were associated reductions in occupational exhaustion. Improved emotional balance was related to reduced depersonalization toward one’s job and colleagues.

This cascade benefit resulting from brain health training being linked to reduced burnout (ratings on questions related to feelings of exhaustion and depersonalization) and improved wellbeing and connectedness on the BrainHealth Index (i.e., higher connectedness, and less mental health complaints of depression, stress, and anxiety) supports our model which conceives brain health as a higher category of health (Chapman et al., 2022). As such, providing individuals with simple steps to build brain skills may serve to improve personalized holistic brain health and attitude toward work. Our prior research has advanced this notion by showing individuals can harness neuroplasticity with tactical brain strategies, that not only improve their neural connectivity, but also their neural health across brain networks in terms of brain blood flow (Chapman et al., 2015; Gallen et al., 2016; Spence et al., 2023).

Training individuals to change their habits with simple steps has shown to promote a sense of self-agency to take charge of one’s own capabilities, drawing upon personal resources to counteract, to some degree, being overwhelmed by external demands of the workplace. This builds a sense of autonomy, one of the main ingredients that helps people thrive in their context (Neill and Nevin, 2021). We propose that realizing one’s role in managing the daily tasks may enhance not only work but may also generalize to one’s personal life, as shown in previous work (Vas et al., 2016; Chapman et al., 2022).

This evidence is consistent with the conservation of resource theory set forth by Hobfoll (1989) and Hobfoll et al. (1990, 2018) suggesting that stressful conditions are rarely single events such as experienced during the pandemic. Rather, the individual response is impacted by the complicated sequences of experiences that emerge over time and the individuals’ perception and reaction to those changes. We propose that the pandemic disrupted almost every aspect of work and personal life, but it was the continual changes that occurred over time that created the dramatic disruption in workplace and home life wellbeing and ability to thrive over time. We propose the current study complements the conservation of resource theory by adding evidence that individuals, when given the mental thinking tools, may be able to counter the powerful bias to over focus on resource loss and instead seek ways to build on resource gain even in the midst of loss and disruption. By engaging in possibility thinking or connecting more closely to team members, individuals’ appraisal of what their options are tends to improve.

### 4.3. Examining how place (WR vs. in-office) impacts brain health and burnout

One unexpected finding was that a balance of days in the office did not have a significant impact on outcomes of holistic BrainHealth Index, factors influencing composite BrainHealth, or burnout components. In fact, we found that fewer days in office were associated with greater gains in the clarity factor, but not for connectedness and emotional balance. We had hypothesized that a hybrid work with 2–3 days in office would provide a ‘sweet spot’ and best ecosystem to support brain health gains as compared to schedules of completely WR or full time in-office, especially following the isolation of the pandemic. A number of corporate leaders believe that organization-wide communication and innovative collaborative work are best achieved by being together in person. Yang et al. (2022) research on Microsoft’s firm-wide shift to complete remote working supports this claim. They found reduced interconnectivity between team members, more stagnant and siloed efforts, an increase in asynchronous communication, and less rich information shared when workforce was WR. Previous work has shown 20–45% to be the sweet spot of time in the office to achieve higher productivity on performance measures (Choudhury et al., 2022).

Several explanations may help explain our failure to find individual-based brain health benefits linked to at least some days in the office in the current study. First is that the finding may be real – that one’s personal ability to thrive may be achievable from any place – home or in-office in or combination of both (Choudhury et al., 2021). Secondly, distinct requirements of positions or responsibilities may allow some to benefit from different working context, e.g., WR versus other job performances are better with In-Office context. The dispersion of patterns could offset individual benefits where no one pattern dominated. Future studies should explore the nature of the job responsibilities and the place of work. Thirdly, we may have failed to find associations between place of work (WR/In-Office) with brain health and burnout measures due to the fact the present study took place at the early transition period of navigating return to office guidelines. As such, considerable flexibility was available in daily choices while still being tracked. Flexibility in choice may be of value to support employee’s wellbeing and sense of agency; however there remains a need for specific guidelines so all are operating with general consensus on expectations. Building brain healthy workplaces may be afforded by a corporate culture where employees have a sense of autonomy in choice as well as the knowledge as to how best to distinctly deploy their mental effort across the range of tasks selecting spaces to optimize performance and connectedness/separateness. The present evidence suggests that deeper level thinking may best be achieved in remote settings. However, remote work may not achieve greater connectedness that is foundational to innovative teamwork and feeling part of the culture with purpose. Evidence suggests some workers are willing to take lower wages in exchange for remote work (Mas and Pallais, 2017), nonetheless, they may miss out on feeling part of the core team effort.

Our findings suggest that there may indeed be some long-term benefits to the hybrid work paradigm that has emerged. For deep work and focus leveraging a space away from the standard open-office layouts may work. Alternately, workplace designers should consider that the workplaces we have today do not support clarity and focus, nor make provisions to support such needs before requiring a return to the office. It is time to consider that the current workplace designs simply do not foster the diverse cognitive demands of the tasks individuals undertake.

Follow-up assessment would help to elucidate whether the lack of association between brain health benefits and average days in the office continues to remain stable over time. Our findings support the view that a variety of options may allow individuals to work toward better brain health and less burnout. Choudhury et al. (2022) show that workplaces that allow intermediate WR arrangements (hybrid) benefit employees almost as much as those who are always in the office in terms of feeling part of a productive and enjoyable workplace. In particular, teamwork may be best achieved with a combination of in-person time and time alone to reflect, innovate and conceptualize better options that comes from both places. We look forward to examining this in future efforts.

### 4.4. Seeking workplace wide trainings to inspire thriving and adaptive employees

As Maslach and Leiter (2021) stated, the COVID-19 pandemic increased burnout and added to workplace distress, leaving organizations in search of ways to address this growing concern. The present findings add to the growing data suggesting that building brain health capacities through trainings may offer ways to buffer the bi-directional effects of burnout and wellbeing in the workplace (Taylor and Millear, 2016). Brain health training via SMART provided the participants with tactical thinking tools, which have previously shown to strengthen the neural networks supporting agility and stability of thought and action, social adeptness, and mental wellbeing, despite challenges. These neural networks purportedly support ability to adapt to rapidly changing contexts of place and purpose (Chapman et al., 2016).

The current work builds on prior work that identified significant predictions between traits of mindfulness and burnout (Taylor and Millear, 2016). Our findings extend prior work on mindfulness to suggest that brain health training protocols positively impacted the individual. Whereas specific mindfulness training was not a part of the SMART protocol, the training included strategies whereby participants were tasked with ‘quieting the mind’ with regular brain breaks, specifically taking 5 – five-minute breaks throughout the day. A brain break specifies that an individual move away from all tasks requiring mental effort to let the brain free up from effortful thinking to allow ideas to free float for ‘aha’ moments of innovative thinking (Chapman et al., 2019). Another key training strategy was for individuals to daily embrace as much change as possible to spark innovation and possibility thinking, since fluidity of ideas has been shown to be a key driver for enhanced brain performance (Chapman et al., 2017, 2019). The brain is wired to adapt to change and gets quickly bored on rote thinking and actions. Being able to continually adapt has shown to reduce stress and enhance wellbeing (Al-Jubari et al., 2022).

### 4.5. Limitations

The present findings must be interpreted cautiously in light of a few limitations. The most important limitation is that the improved individual brain health outcomes do not address whether the individual gains could be linked to a productivity and economic benefit to the organization. The bottom-line of economic gain matters. Future efforts are needed to explore how taking a human-centric approach that promotes the individual’s brain health and performance influences the ROI important to employers such as retention and attraction of talent, productivity, engagement, reduced medical health expenses, fewer days absent, to mention a few. Nonetheless, the present findings represent a concerted effort to show positive benefits when an organization puts the individual’s wellbeing over and above their own bottom line.

A second limitation is attrition and the lack of longer-term follow-up to see whether continually reinforcing brain health strategy deployment will contribute to persistent and even greater gains in brain health at an individual and organizational level. We are continuing to study the prolonged effects of the brain health trainings both to the individual and to teamwork and collaborative efforts when they share a common language related to brain healthy habits to hold each other accountable. Based on best practices recommendations, we are sharing the results of this study with the participants to garner their input regarding lessons learned and to adjust what seems to work best for the teams. We believe this will help individuals remain curious about the degree to which they can improve their brain health and seek different ways to maintain or enhance their current brain skills to stay top of their mental game. For example, they may focus on improving *connectedness* to either the people or the purpose of work, or better *clarity* in expanding possibility thinking to solve problems faced day to day.

A third limitation is the high variability among different offices within the single organization in terms of work environment and culture and lack of knowledge about how much the agency employees had in selecting WR versus in-office modes of working. At the time of this study, the organization was undergoing changing policies about presence in the office. Whereas we were able to track the average number of days working remote versus in-office, we did not have access to individual choice, nor those for whom the days in office were being set for them. Some experts have suggested that if an individual is given agency in the decision about place of work, it may benefit the individual and the organizational outputs. In our ongoing research, we hope to address whether free choice or declared policy influences brain health and performance and aspects contributing to burnout. Flexibility in choice as well as having a stated expectation may be important to help an employee’s ability to thrive.

A fourth limitation is that recruitment was from a voluntary pool of individuals who had completed at least one firm-initiated pulse survey. The leadership encouraged participation among this group but agreed not to have any access to either knowing who participated or any individual results to alleviate concern the participants may have held as to how data/results were being used. Those who chose versus those who did not choose to participate may have had very different results. A related limitation was whether some differences might exist between those who completed their T2 assessment and those who did not. However, both groups were found to be comparable on our baseline measures, suggesting no meaningful differences. The failure to complete the second assessment may be due to a variety of individual issues such as time, motivation, fear of no gain and even perhaps losses, to mention a few. Future work could add surveys to capture individual reasons for lack of completion and fuller engagement.

Lastly, we acknowledge that the lack of a control group may limit the perceived level of evidence from this study to support training benefits. Individuals may have improved over the 6-month interval even without the brain health training due to any number of reasons such as conditions in the world began to improve or self-adjustments to the new order of work. Whereas these could certainly be factors, the evidence that individuals who completed the most training modules improved the most support our claim that the training contributed to the gains as those who did little to no training failed to show significant gains. These gains are similar to our prior study of dispersed individuals showing a significant training dose effect such that those who took advantage of more of the training modules showed greater gains (Chapman et al., 2021). Based on similar prior results, we feel the gains in the present study were likely due to training utilization. Additional support that our single arm trial evidence is informative is that previous randomized clinical trials have found significant gains from the brain health trained group with limited gains in the active control groups (Chapman et al., 2015; Vas et al., 2016; Chapman et al., 2017).

It is also important to note that this study was executed with an organization that was interested in a field study to test real world application given the consistent existing clinical trial evidence that the brain health program showed benefits. The firm was less interested in a randomized control trial and more so in the degree to which employees would take advantage of the offering and the resulting impact.

## 5. Conclusion and future possibilities

The present study represents an early attempt to examine how an online training centered around individual capacity-building principles of neuroplasticity, combined with personalized coaching would improve brain health, reduce aspects of burnout, and interact with remote versus in-office contexts. In the past, many employers have offered programs to identify and address mental health concerns, such as stress with mindfulness and depression, and other diagnoses. We propose an alternate, complementary approach that seeks to build potential and benefit *across all employees* to supplement those that primarily seek to address medical concerns.

The findings provide clear evidence that a majority of individuals across the age range, job position, experience level, and gender were able to reap benefits from the brain health trainings. The improvement in overall brain performance as well as in all three factors (i.e., clarity, connectedness and emotional balance) and the associated reductions in emotional exhaustion and depersonalization toward the job provides compelling evidence that the gains impacted improved feelings toward work and the mental demands. The unexpected finding of improved clarity with fewest average days/week in the office suggests that place of work perhaps should be calibrated to match task demands. Individual factors such as job responsibilities and team interactions required to perform as well as demands of the home remain critical factors to consider.

This work is particularly important because it shows that organization-wide trainings that benefit an individual’s growth and capacity-building may be important in the future of work. Today, workers are more than ever interested in work-life balance and are seeking ways to thrive, cognitively, socially, emotionally, and physically not only in their work but maybe even more importantly in their personal life before burnout and mental health issues become significant issues.

Whereas many unknowns exist as to how the future of work will be designed, one proposition that is receiving growing acceptance is that a company that *focuses on individuals first* – as a human centric approach above measuring corporate productivity as the top goal—will likely reap spillover rewards to the workplace bottom line. This study provides promising evidence that focusing on the individual first has its rewards. Now efforts are needed to address the degree to which individual gains benefit the organization as a whole. Futuristically, we are pushing the frontier to determine whether better brain health will have significant economic benefits that span from the individual to their work and community to drive positive impact of global wellbeing - which we named *Brainomics* (Chapman, 2013). Brainomics seeks to quantify the overall economic impact of improved brain health and performance.

Reports reveal that the younger generation of workers is welcoming the profound change that has taken place in working patterns of place, speaking out about their desire for a better work-life balance than previously existed– not just to succeed at work and climb the corporate ladder. Employers are in a quandary regarding what is best for achieving corporate goals while continuing to attract and retain top talent. By 2025, more than a fourth of the workforce will be Gen Z or people born between 1997 and 2012. Gen Z workers have changing needs with different workforce design demands than worked in the past. They want to have greater sense of autonomy, flexibility and programs that provide tools to better navigate workplace and personal desires, allowing them to thrive and make an impact on the world (Abril, 2022). The optimal formula for WR and In-Office combinations remain elusive and may be individually determined based on benefits and drawbacks. What is clear is one-size-does-not-fit all and focusing on measurement and training to give individuals the tools to build and draw upon individualized brain skills may help guide decisions.

In conclusion, the workplace is in a time of profound period of reshaping and transformation. Companies are realizing they need to invest in their existing workforce. Boosting brain skills is key to a thriving workforce as well as to human-centered fulfillment of living a good life (Greene, 2021; Smith et al., 2021). These early findings support a perspective that both are possible as individuals learn the tools to strengthen their composite brain health performance by prioritizing connectedness, clarity and emotional balance. The emerging science of brain health is showing that each person can take charge of our own brain health, similar to what has been achieved for heart health. By focusing on brain health as a higher category of health promotion – we can start to change the return-to-office conversation away from a primary focus on measuring productivity and a laser focus on mental health problems to one of exploring ways to promote the individual’s ability to thrive.

## Data availability statement

The raw data supporting the conclusions of this article will be made available by the authors, without undue reservation.

## Ethics statement

The studies involving humans were approved by The University of Texas at Dallas Institutional Review Board. The studies were conducted in accordance with the local legislation and institutional requirements. The participants provided their written informed consent to participate in this study.

## Author contributions

JZ contributed to study design, performed oversight of assessment and training execution, collected and scored data, provided participant coaching, wrote portions and edited the manuscript. JS organized the database, performed statistical analysis, and wrote portions of the manuscript. SuC contributed to study design, performed oversight of engagement, and contributed to the manuscript. UN contributed to study design, contributed to the manuscript, and performed oversight of engagement. SaC contributed to study design, interpreted results, and wrote the manuscript. All authors contributed to manuscript revision, read, and approved the submitted version.

## Funding

This research was made possible thanks to The Baldridge Foundation, Jean Ann Brock, The Joshua M. and Inette S. Brown Family Foundation, Peggy Dear, Estate of Alice Janet DeSanders, Teresa and David Disiere, Folsom Charitable Foundation, Jane and Mark Gibson, The Carlos and Deborah Hernandez Foundation, Kozmetsky Family Foundation, The J. Willard and Alice S. Marriott Foundation, J. Willard Marriott, Jr. Foundation, Shirley & William S. McIntyre Foundation, Ellen and John McStay, Marlane Miller, Laurie and Todd Platt, Jennifer and Peter Roberts, Sammons Enterprises, Inc., the Dee Wyly Distinguished University Chair for BrainHealth, as well as several anonymous donors. The authors declare that this study received funding from the Sammons Enterprises, Inc. The funders were not involved in the study design, collection, analysis, interpretation of data, the writing of this article or the decision to submit it for publication.

## Conflict of interest

SuC and UN are employed by HKS Inc.

The remaining authors declare that the research was conducted in the absence of any commercial or financial relationships that could be construed as a potential conflict of interest.

## Publisher’s note

All claims expressed in this article are solely those of the authors and do not necessarily represent those of their affiliated organizations, or those of the publisher, the editors and the reviewers. Any product that may be evaluated in this article, or claim that may be made by its manufacturer, is not guaranteed or endorsed by the publisher.
